# Correspondence from New York

**Published:** 1864-12

**Authors:** 


### CORRESPONDENCE.

NEW YORK, DEC. 20, 1864.

DR. DAVIS,—*My Dear Sir:*

According to promise, I drop you a line concerning professional matters, in some of our Eastern cities. On my way to New York, I made a short stay of one week in Philadelphia. I found the medical schools in active operation, with a respectable class of students in attendance. The matriculants for this term in the University of Pennsylvania numbered (Nov. 10th) a little over 300; in the Jefferson about the same number. The changes which have occurred recently in the Faculty of these Schools are as follows:—The vacancy in the chair of Practice in the University, caused by the resignation of Dr. Pepper, who was the successor of Dr. Geo. B. Wood, is filled by Prof. Alfred Stille. The chair of Obstetrics, made vacant by the resignation of Dr. Hodge, is filled by Prof. Penrose: Prof. P. is a young man less, I judge, than 40 years of age. In the Jefferson, the chair of Chemistry, which the death of Prof. Bache left vacant, is filled by Prof. B. Howard Rand, whom I judge to be about 40 years of age, and the youngest man in the Faculty of Jefferson.

Of the two schools the University (which is in its ninty-ninth Session) is the most popular hereabouts; while the class in Jefferson is mainly made up of students from the Western States. Six lectures are delivered in each per day, and the same chairs are represented at usually the same hour. The clinical instruction is mainly given at Blockley Hospital, which is three miles away from either college. The Faculty of the University have the service and deliver the clinics during the first half of the college term; after which the same duty is performed by the Faculty of Jefferson. Dr. Agnew has the first period of surgical service, and is followed by Prof. Gross.

These venerable schools of medicine afford excellent didactic lectures to their classes; but certainly they are far behind the present demands of the profession in that kind of instruction calculated to fit the student for practice. I refer to clinical. To be sure, two clinics are given a week in Blockley and Pennsylvania Hospitals, in which are found an abundance of morbid material. But the cases are all brought into the amphitheatre, in the presence of 400 or 500 students, not one out of every twenty is any the wiser for the presence of the patient; scarcely one-half can tell from their seats whether the patient is an African or a Celt. And to see a surgical operation is simply out of the question.

It is, however, a pleasure to visit, for a short time, these ancient schools of the prophets, to see and hear the medical patriarchs, Drs. Dunglison, Gross, Pancoast, Dickson, and others, whose works do honor to our medical literature.

The medical schools of New York I found all busy, except the New York Medical College, on 13th Street, which has died from the want of support. The institution had never paid, except *out of pocket.* The college building is now being made into a masonic hall.

The University Medical College continues to progress on the even tenor of its way, having, at present, a class of about 100 students. Prof. Metcalf, of the chair of Practice, has mainly withdrawn from the teaching, on account of ill-health and domestic affliction; his place being filled by Dr. Loomis. Prof. Budd holds the chair of Obstetrics, vacated by the resignation of Prof. Bedford. Prof. Mott, Sen., lectures occasionally.

The College of Physicians and Surgeons continues to maintain its former popularity, having a class of 250 students. Prof. T. G. Thomas fills the chair of Obstetrics formerly occupied by Prof. Gillman.

The Bellvue Medical College has a class of about 300 matriculants—a class too large for its present accommodations. But a new building is in process of erection by the Commissioners of Public Charities, of ample dimensions. It is to be 60 by 100 feet, five stories high. The first floor is for a dispensary for out-patients. The lecture-rooms—two in number—are to be capable of seating 600 students. It is located adjoining and west of the present building, which is to be converted into a museum for the College. The success of this school is remarkable, giving an argument, at last, that the old rotinue of medical instruction is not invulnerable—and not to last forever.

The mcst important of the medical societies of New York are the Academy of Medicine and the Pathological Society, which meet every Wednesday evening alternately. These societies are participated in by the very best medical talent in the city.

At a meeting of the Academy (Nov. 16th), the subject for discussion was:—"The Ulterior Physiological and Pathological

Effects of Anesthetics." The discussion was opened by Dr. Frank Hamilton, whose views were new and startling. He said, in substance, that he believed anesthetics had produced ten times as many deaths as was generally believed, especially in the army; that they injured the blood; often producing alarming and dangerous prostration and vomiting; they rendered the muscular flaps in capital operations flabby and lifeless for 24 to 48 hours; and that it was rarely possible to get union by the first intention after their use. He cited Drs. Mott, Wood, and Post, as authority, that at an early period, before anesthetics were used, union by the first intention was the almost invariable rule, after capital operations, throughout the City and in the New York Hospital. And in the army, where anesthetics were used so freely, the surgeons almost universally testified, that after amputations union without suppuration was impossible; and, finally, abundant proof could be adduced, that the same results obtained, he believed from the same cause, in the army of the Crimea.

These views were combated by several members. Dr. Detmold said, it was well known that in our armies a scorbutic diathesis had existed, which prevented good success in surgical operations. That this diathesis existed in the Crimean army was a matter of history. He argued, that at an early day, when the city of New York was comparatively small, and the N. Y. Hospital smaller and less crowded than of late years, better success, of course, was obtained in surgery—the reason was manifest. He believed the physiological effects of anesthetics, though powerful for a short time, were temporary—soon leaving the patient in as favorable condition as before their administration. Dr. Connant, and others, advanced similar views. Dr. Squibb said, he thought much of the injury from anesthetics was the induction of asphyxia from too great exclusion of atmospheric air, as well as syncope from too long administration of the remedy. Notwithstanding the opposition excited by Dr. Hamilton's remarks, the logical manner in which he advanced his views, and the interesting facts which he had collected from his extensive observations in military and civil hospitals, secured to him the respectful attention of the Academy, and impressed many of the members with the soundness of his opinions on this subject.

A new medical society was recently organized here, through the efforts of Dr. Isaac E. Taylor, called the "Medical Journal Association." They propose to fit up a reading room, and furnish it with all the leading medical journals of this country and Europe. The room is to be kept constantly warmed, lighted, and in the care of a janitor. A full corps of officers are elected. Dr. Delafield, president, and Dr. Noeggerath, corr.-secretary. The profession of the City is very generally interested in this movement.

Medical journalism in New York, at present, is at a low ebb. However, rumor whispers that medical journals, from two or three different combinations, are soon to appear. The *Medical Times*, too, is to be revived, whether in a weekly or a monthly form deponant answereth not. But more anon. R. P. J.

